# Supplementary material for: Implementing supportive supervision in acute humanitarian emergencies: Lessons learned from Afghanistan and Ukraine
Source: PLOS Glob Public Health. 2024 Apr 17;4(4):e0002416. doi: 10.1371/journal.pgph.0002416 (PMC11023585; doi:10.1371/journal.pgph.0002416)
Supplement: Attachment — Submitted filename: Response to reviewers.docx [file pgph.0002416.s002.docx]

Response to reviewers:

We would like to express our sincere appreciation for our two reviewer’s insightful and helpful comments and suggested modifications for our proposed manuscript. In the sections below, we have addressed each comment and incorporated the recommended changes throughout the text of our paper. The changes are in track changes in the text of the manuscript for ease of identification.

Journal Requirements:

1. Please review your reference list to ensure that it is complete and correct. If you have cited papers that have been retracted, please include the rationale for doing so in the manuscript text, or remove these references and replace them with relevant current references. Any changes to the reference list should be mentioned in the rebuttal letter that accompanies your revised manuscript. If you need to cite a retracted article, indicate the article’s retracted status in the References list and also include a citation and full reference for the retraction notice.

**Response**: Reference list was reviewed and no changes were required.

2. Please include the following request in the decision letter, and ping me with follow-up. “Please include a complete copy of PLOS’ questionnaire on inclusivity in global research in your revised manuscript. Our policy for research in this area aims to improve transparency in the reporting of research performed outside of researchers’ own country or community. The policy applies to researchers who have travelled to a different country to conduct research, research with Indigenous populations or their lands, and research on cultural artefacts. The questionnaire can also be requested at the journal’s discretion for any other submissions, even if these conditions are not met.  Please find more information on the policy and a link to download a blank copy of the questionnaire here: <https://journals.plos.org/globalpublichealth/s/best-practices-in-research-reporting>. Please upload a completed version of your questionnaire as Supporting Information when you resubmit your manuscript.”

**Response:** Completed and uploaded, as requested.

3. In the online submission form, you indicated that "Data can be made available by the author upon reasonable request". All PLOS journals now require all data underlying the findings described in their manuscript to be freely available to other researchers, either 1. In a public repository, 2. Within the manuscript itself, or 3. Uploaded as supplementary information.
This policy applies to all data except where public deposition would breach compliance with the protocol approved by your research ethics board. If your data cannot be made publicly available for ethical or legal reasons (e.g., public availability would compromise patient privacy), please explain your reasons by return email and your exemption request will be escalated to the editor for approval. Your exemption request will be handled independently and will not hold up the peer review process, but will need to be resolved should your manuscript be accepted for publication. One of the Editorial team will then be in touch if there are any issues.

**Response:** We have replied to your email as requested (please see email from Nadeen Abujaber dated August 23^rd^, 2023).

Reviewers' comments:

Reviewer's Responses to Questions

**Comments to the Author**

1. Does this manuscript meet PLOS Global Public Health’s [publication criteria](https://journals.plos.org/globalpublichealth/s/criteria-for-publication)? Is the manuscript technically sound, and do the data support the conclusions? The manuscript must describe methodologically and ethically rigorous research with conclusions that are appropriately drawn based on the data presented.

Reviewer #1: Yes

Reviewer #2: Yes

2. Has the statistical analysis been performed appropriately and rigorously?

Reviewer #1: N/A

Reviewer #2: N/A

3. Have the authors made all data underlying the findings in their manuscript fully available (please refer to the Data Availability Statement at the start of the manuscript PDF file)?

The [PLOS Data policy](https://journals.plos.org/globalpublichealth/s/data-availability) requires authors to make all data underlying the findings described in their manuscript fully available without restriction, with rare exception. The data should be provided as part of the manuscript or its supporting information, or deposited to a public repository. For example, in addition to summary statistics, the data points behind means, medians and variance measures should be available. If there are restrictions on publicly sharing data—e.g. participant privacy or use of data from a third party—those must be specified.

Reviewer #1: Yes

Reviewer #2: No

4. Is the manuscript presented in an intelligible fashion and written in standard English?

PLOS Global Public Health does not copyedit accepted manuscripts, so the language in submitted articles must be clear, correct, and unambiguous. Any typographical or grammatical errors should be corrected at revision, so please note any specific errors here.

Reviewer #1: Yes

Reviewer #2: Yes

5. Review Comments to the Author

Please use the space provided to explain your answers to the questions above. You may also include additional comments for the author, including concerns about dual publication, research ethics, or publication ethics. (Please upload your review as an attachment if it exceeds 20,000 characters)

Reviewer #1: This is a nicely written manuscript that highlights an important problem and a relevant intervention. The main limitation is the small sample but this is understandable. Would it be possible to know more about the participants besides role and gender without breaching confidentiality? Years of experience in the field, nationality etc. This would be helpful to contextualize their answers.

**Response:** Thank you for your suggestions. We have added the following information about participants (where available) in Table 1: years of experience in MHPSS, years providing supervision and years receiving supervision. We were concerned, however, that providing additional information like nationality (particularly for participants operating as international staff), age, or professional title would risk breaching confidentiality and may even pose a security risk to our participants, so have omitted these. Please see page 6 of the manuscript for the revised table.

Given the importance, I would have liked more references and emphasis in the discussion and conclusion to the problem itself outside the confines of the organization and the specific intervention. Basically how can other organizations in the humanitarian field learn from it or use it.

**Response:** Included as suggested. Please see section ‘Practical Implications’, page 26, lines 598-631 and ‘conclusion’, page 27, line 631.

Otherwise I believe it is an interesting paper that deserves publication

Reviewer #2: 1. This is an action research and the authors have presented data from the interviews of five participants.
It was however not clear if all five participants were also involved in a group discussion. The authors used the word 'discussed' while presenting the results. See for example lines 214, 236, 253 282. Themes and sub- themes are appropriate and relevant.

**Response:** Participants were not involved in group discussion. The word ‘discussed’ was describing the discussion between each individual participant and the interviewers. We have clarified this and made changes where appropriate in the manuscript (please see page 9, line 218; page 10, line 240; page 11, line 258; page 12, line 273; page 15, line 347; page 16, line 374; page 17, lines 393 and 401; page 18, line 434 and page 21, lines 489, 499 and 505)

2. The study being a qualitative one did not require statistical analysis.
3. The authors mentioned that the availability of the data will be upon request during to the sensitivity of the topic under study
