# Supplementary material for: Implementing supportive supervision in acute humanitarian emergencies: Lessons learned from Afghanistan and Ukraine
Source: PLOS Glob Public Health. 2024 Apr 17;4(4):e0002416. doi: 10.1371/journal.pgph.0002416 (PMC11023585; doi:10.1371/journal.pgph.0002416)
Supplement: Attachment — Submitted filename: Response to Reviewers1.docx [file pgph.0002416.s003.docx]

Response to Reviewers:

Sincerest appreciation for our reviewer’s comments and suggestions. We have addressed the recommended changes within the text of manuscript as described below. The changes are in track changes in the text of the manuscript for ease of identification.

___________________________________________________________________________________________

The reviewers have agreed that this paper is technically sound and should be considered for publication. A few small comments to address first.
Reviewer 1
1. There are couple of grammatical errors and would advise the authors to check for all long sentences that are unclear - example - lines 99, 100, lines 104 -108, lines 108 - 112.

**Response to Reviewers: Corrected.**

Reviewer 2
1. Ethics approval clarification - "It will be interesting to know if the ethical approval from Trinity College Dublin Health Policy and Management /Centre for Global Health ethics committee which is not based in the countries where data was collected, is valid for this study."

**Response to Reviewers: Since the authors were unable to obtain ethical approval from both countries given the acute humanitarian crises and since data was being collected (remotely) in Ireland (and not in-country in Afghanistan or Ukraine), ethical approval from Trinity College Dublin Health Policy and Management /Centre for Global Health ethics committee in Ireland was deemed valid.**

2. proofing "There is an omission to italicize a t on line 299 on the reported speech of a participant in the study."

**Response to reviewers: Corrected.**

Journal Requirements:

1. Please include a complete copy of PLOS’ questionnaire on inclusivity in global research in your revised manuscript. Our policy for research in this area aims to improve transparency in the reporting of research performed outside of researchers’ own country or community. The policy applies to researchers who have travelled to a different country to conduct research, research with Indigenous populations or their lands, and research on cultural artefacts. The questionnaire can also be requested at the journal’s discretion for any other submissions, even if these conditions are not met.  Please find more information on the policy and a link to download a blank copy of the questionnaire here: <https://journals.plos.org/globalpublichealth/s/best-practices-in-research-reporting>. Please upload a completed version of your questionnaire as Supporting Information when you resubmit your manuscript.

**Response to reviewers: Already completed as part of the previous revision process.**

Please review your reference list to ensure that it is complete and correct. If you have cited papers that have been retracted, please include the rationale for doing so in the manuscript text, or remove these references and replace them with relevant current references. Any changes to the reference list should be mentioned in the rebuttal letter that accompanies your revised manuscript. If you need to cite a retracted article, indicate the article’s retracted status in the References list and also include a citation and full reference for the retraction notice.

**Response to reviewers: Reviewed and no changes identified.**
